# Effects of the Use of Automatic Tube Current Modulation on Patient Dose and Image Quality in Computed Tomography

**DOI:** 10.4274/mirt.galenos.2019.83723

**Published:** 2019-09-06

**Authors:** Ayşegül Yurt, İsmail Özsoykal, Funda Obuz

**Affiliations:** 1 Dokuz Eylül University Faculty of Medicine, Department of Medical Physics, İzmir, Turkey; 2 Dokuz Eylül University Faculty of Medicine, Department of Radiology, İzmir, Turkey

**Keywords:** Tomography, radiation protection, abdomen

## Abstract

**Objectives:**

The frequency of abdominal computed tomography examinations is increasing, leading to a significant level of patient dose. This study aims to quantify and evaluate the effects of automatic tube current modulation (ATCM) technique on patient dose and image quality in contrast-enhanced biphasic abdominal examinations.

**Methods:**

Two different scan protocols, based on constant tube current and ATCM technique, were used on 64 patients who visited our radiology department periodically. For three patient groups with different patient size, results from two protocols were compared with respect to patient dose and image quality. Dosimetric evaluations were based on the Computed Tomography Dose Index, dose length product, and effective dose. For the comparison of image qualities between two protocols, Noise Index (NI) and Contrast to Noise Ratio (CNR) values were determined for each image. Additionally, the quality of each image was evaluated subjectively by an experienced radiologist, and the results were compared between the two protocols.

**Results:**

Dose reductions of 31% and 21% were achieved by the ATCM protocol in the arterial and portal phases, respectively. On the other hand, NI exhibited an increase between 9% and 46% for liver, fat and aorta. CNR values were observed to decrease between 5% and 19%. All images were evaluated by a radiologist, and no obstacle limiting a reliable diagnostic evaluation was found in any image obtained by either technique.

**Conclusion:**

These results showed that the ATCM technique reduces patient dose significantly while maintaining a certain level of image quality.

## Abstract (tr)

**Amaç:**

Abdominal bilgisayarlı tomografi incelemelerinin sıklığı artmakta ve önemli miktarda hasta dozuna yol açmaktadır. Bu çalışma, kontrastlı, çift fazlı abdomen incelemelerinde otomatik tüp akım modülasyon tekniğinin hasta dozu ve görüntü kalitesi üzerine etkilerini ölçmeyi ve değerlendirmeyi amaçlamaktadır.

**Yöntem:**

Radyoloji anabilim dalını periyodik olarak ziyaret eden 64 hastaya sabit tüp akımı ve otomatik tüp akımı modülasyonu tekniğine dayanan iki farklı tarama protokolü uygulandı. Farklı hasta boyutlarına sahip üç hasta grubu için, iki protokolden elde edilen sonuçlar hasta dozu ve görüntü kalitesi açısından karşılaştırıldı. Dozimetrik değerlendirmeler, Bilgisayarlı Tomografi Doz İndeksi, doz uzunluk çarpımı ve etkin doza dayandırıldı. İki protokol arasındaki görüntü kalitesinin karşılaştırılması amacıyla her görüntü için Gürültü İndeksi (NI) ve Kontrast Gürültü Oranı (CNR) değerleri belirlendi. Ek olarak, her görüntü deneyimli bir radyolog tarafından öznel olarak değerlendirildi ve sonuçlar iki protokol arasında karşılaştırıldı.

**Bulgular:**

Otomatik tüp akım modülasyon protokolü ile arteriyel ve portal fazlarda sırasıyla %31 ve %21 doz düşüşleri sağlandı. Öte yandan, NI karaciğer, yağ ve aort için %9 ile %46 arasında bir artış gösterdi. CNR değerlerinin ise %5 ile %19 arasında azaldığı gözlendi. Tüm görüntüler bir radyolog tarafından değerlendirildi ve herhangi bir teknikle elde edilen görüntülerde güvenilir bir tanısal değerlendirmeyi sınırlayan bir engel bulunmadı.

**Sonuç:**

Bu sonuçlar, otomatik tüp akım modülasyon tekniğinin, belirli bir görüntü kalitesi seviyesini korurken hastanın dozunu önemli ölçüde azalttığını göstermiştir.

## Introduction

In the early 1990s, helical computed tomography (CT) devices were introduced for medical imaging. Shortened examination times, improved visibility of vascular structures and potential reduction in the use of contrast material enabled intensive use of this technology. However, the clinical use of CT increased mainly after multislice helical CT scanners became available towards the end of the decade. Today, images from 64 to 320 slices can be acquired in a single rotation of the X-ray tube within one-third of a second. These advances led to a further increase in the use of CT for cardiovascular examinations, perfusion imaging, brain, heart, breast, colon, and whole body studies (1).

Radiation exposure of patients having CT scans has increased as a consequence of more frequent use of CT. Recent studies on major medical centers in the UK showed that only 11% of all applications in the radiology departments are CT applications, whereas the effective radiation dose of patients due to CT applications was reported as 40% in 1998 and 68% in 2008 (2). Although offering shorter image acquisition time and higher spatial resolution, multislice CT technology has some dosimetric handicaps to be considered. In MSCT, over-beaming and end effect terms refer to the necessity of beam and scan widths extending beyond detector area and imaged region, respectively. These conditions that arise due to image reconstruction purposes lead to increase in radiation dose to the patient, when compared to single slice CT scanners. On the other hand, smaller gantry designs for MSCT devices led to a shorter patient-tube distance which obviously affects patient dose (3). These conditions have forced CT manufacturers to develop dose optimization strategies either based on image processing or the prevention of unnecessary radiation. The most common strategy among these is the use of Automatic Exposure Control (AEC), where the tube current is adjusted by the scanner according to the patient size. Since the beginning of the 2000s, AEC systems have been developed by the manufacturers based on different operating mechanisms; however, offering similar opportunities on patient dose control, image quality, and tube life (4,5).

In CT, AEC is applied based on two main techniques: Automatic Current Setting (ACS) and Automatic Tube Current Modulation (ATCM), which can be activated separately or combined. In ACS technique, scanner generates an optimized constant tube current to be applied along the scanned region for which ATCM offers a modulated tube current. This modulation may be achieved either for every single longitudinal slice along the z-axis or at different angular projections of the tube on x-y plane. These techniques are known as longitudinal ATCM and angular ATCM, respectively.

Longitudinal ATCM, a commonly used ACS technique, is available under different names among different manufacturers. Z-DOM, a longitudinal ATCM named by Philips, makes use of a pre-scan radiograph, named as a topogram, to compute the attenuation properties of the patient as a function of scan length and modulate tube current based on this information. This dose modulation mechanism works in accordance with a reference image quality selected and standardized by the user, in terms of a Noise Index (NI) (6). In CT exams that include both head&neck and abdominal regions, for example, Z-DOM technique achieves both radiation protection in thyroids and good image quality in abdominal region by locally decreasing and increasing tube current. However, scan protocols applying constant tube current usually fail to meet these goals at the same time. These scans end up with either overexposure of thyroids or underexposure of abdominal region depending on the amount of tube current.

In the literature, studies carried on the abdominal CT examinations of adults report commonly that the use of AEC techniques leads to a considerable decrease in patient dose while keeping a reasonable image quality (6,7,8,9,10,11,12,13,14). This study aims to focus on the use of Z-DOM in contrast-enhanced biphasic abdominal examinations and to make evaluations on image quality and patient dose. The results will be examined with respect to different patient groups in different size.

## Materials and Methods

### Patient Profile and Scan Protocol

This retrospective study was conducted in accordance with ethical standards under the responsibility of the Institutional Review Board that approved the study (decision no: 2015/05-19). Sixty four patients undergoing contrast-enhanced biphasic abdominal CT examination were involved in the study. The scans were performed with a 64-slice CT scanner (Brilliance, Philips medical systems, Netherlands) which is located in the radiology department of our university hospital. All data regarding both image quality and dosimetric quantities were classified under three patient groups with respect to patient size for a better evaluation of the results. This classification was carried out based on CT images, according to the effective diameter measurements of the patients taken from the abdominal region. Effective diameter, D_eff_, was determined using lateral and anterioposterior sizes of the patient as shown in Equation 1.

D_eff_ = (D_LAT_ + D_AP_)/2                      (1)

Patients with effective diameters in the range of 21-26 cm were included in the first group, patients with effective diameters in the range of 26-31 cm were included in the second group, and patients with effective diameters in the range of 31-36 cm were included in the third group.

**Figure 1:**
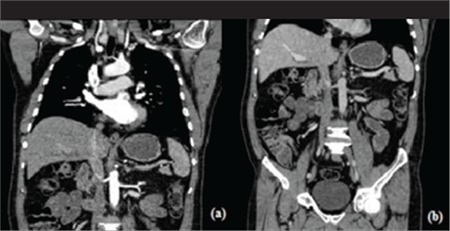
Scan regions for arterial phase (A) and portal phase (B)

Cohort of the study involved the patients who underwent biphasic abdominal examinations periodically. In these examinations, the arterial phase scan involved thorax and abdomen while portal phase scan involved abdominopelvic region (Figure 1). Scan parameters regarding weight based routine protocol and ATCM protocol are given in Table 1. All parameters were kept constant except effective tube current.

### Patient Dosimetry

Computed Tomography Dose Index (CTDI_vol_) and Dose Length Product (DLP) values are two main dosimetric quantities reported by the scanner following each exam. CTDI_vol_ refers to the dose output of the CT scanner measured in a cylindrical PMMA phantom with an ionization chamber. It represents absorbed dose, in mGy, in the central slice of the scan range. Therefore, it is not a direct measure of patient dose, however, it offers the opportunity for dosimetric comparison between different scanning protocols and it is commonly used for quality control purposes. DLP, on the other hand, represents the total radiation output of a scanner along the axis of scan and it is determined by multiplying CTDI_vol_ with the scan length. These two quantities were obtained from examination specific dose reports given by the scanner which has been objected to a dosimetric quality control test prior to the collection of data. Besides CTDI_vol_ and DLP, effective dose (E) was calculated for each scan using E per DLP (E/DLP) value recommended by the European Commission’s Guidelines, as shown in Equation 2.

E = E_DLP_ x DLP                  (2)

Here, E stands for the E (mSv) to the patient due to CT scan. E_DLP_ represents the E per DLP, and it is given as 0.015 mSv/mGy.cm specific to abdominal scans (15). Two scan protocols were compared based on CTDI_vol_, DLP and E.

### Image Quality

In this part of the study, following the dosimetric comparison, NI and Contrast to Noise Ratio (CNR) of the images obtained via both protocols were compared, and the image quality was examined objectively based on these parameters as recommended by the international authorities. In addition to this, subjective evaluation made by a clinician was another method in which image quality was considered.

### Objective Approach

**Figure 2:**
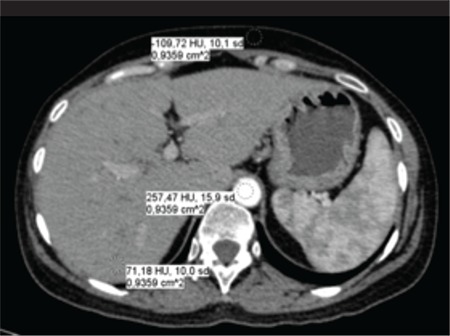
Objective analysis of image quality

Objective analysis of the image quality was based on NI which is defined as the standard deviation in the pixel values (i.e., Hounsfield Units, HU) for a homogeneous object being scanned. Circular region of ınterest (ROI) was drawn to measure NI in three regions: The subcutaneous fat in the anterior region of the abdomen, liver, and aorta. Figure 2 shows three ROIs with identical areas that were cared to be located at the same regions for each patient. For each image, an average NI calculation was made based on the NI measurements taken in three consecutive slices. Apart from NI, average CNR values were determined to compare the images by means of contrast resolution. CNR value of two tissues A and B was determined as shown in Equation 3 (16):

CNR = (S_A_-S_B_) / [(SD_A_)^2^ + (SD_B_)^2^]^1/2^               (3)

Where S_A_ and S_B_ denote mean HU values within the ROIs while SD_A_ and SD_B_ denote the standard deviation, or NI, measured for tissues A and B, respectively. CNR values were obtained for liver-fat and aorta-fat and compared between two scan protocols (Figure 2).

### Subjective Approach

In addition to the objective analysis of image quality, subjective evaluations were made on images by a radiologist who rated the overall image quality and the visibility of anatomic details. This evaluation was done by grading the diagnostic quality of the image examined without any information known about the scan protocol. The grading scale is given in Table 2. Minimum grade required for an image to be regarded as acceptable in terms of diagnostic quality was determined as 2, referring to a study carried out by Mulkens et al. (14).

### Presentation and Statistical Analysis of Data

Among all data obtained for patient dose and image quality, arithmetic mean values were calculated and presented for different patient groups (1, 2 and 3) as well as all patients (overall). Besides, data obtained for dosimetric and objective image quality purposes were analyzed statistically using Mann-Whitney U and t-test, respectively.

## Results

In this study, 30 female and 34 male patients were examined. The mean age of the patients was 57.4±12.7 years. On the other hand, mean D_eff _values were found to be 23.8±2 cm, 28.9±1.4 cm, and 33.1±1.5 cm for group 1, group 2 and group 3, respectively. In Table 3 and Table 4 are given the dosimetric results obtained for each biphasic scan protocol for different patient sizes. ATCM protocol was observed to lead 31% and 21% reductions in E for arterial and portal phases, respectively, according to the results obtained from all patient groups, as given in Table 5 which also represents the results based on different patient groups.

### Statistical Analysis

Statistical analysis on dosimetric data mostly gave significantly different (p<0.05) results across patient groups for routine and Z-DOM scanning protocols. The only exception was the portal phase examination of patients in group 3 for which the dosimetric results were not observed to be significantly different for two scan protocols (p>0.05).

Findings based on NI and CNR obtained for the objective image quality comparison of two protocols are given in Table 6. As observed, images obtained from the ATCM protocol had higher NI and lower CNR values compared to the routine protocol. However, statistical analysis showed that, for patients in group 3, there was no significant difference between two protocols based on NI and CNR values (p>0.05), unlike the findings obtained from group 1 and group 2 (p<0.05).

Results from subjective image quality evaluations made by a radiologist are given in Table 7. Findings indicated that all images met an acceptable level of diagnostic quality, regardless of which scan protocol was used.

## Discussion

Patient size, institution-specific scan protocols and the use of multiphase scanning are three main factors that affect patient dose in CT examinations (17). In this study, a new scan protocol that used Z-DOM was evaluated against routinely used constant tube current protocol for biphasic abdominal CT exams. The two quantities of evaluation were patient dose and image quality.

Z-DOM protocol was observed to lead significant reductions in CTDI_vol_, DLP and E values across all patient groups (Table 3 and Table 4). The percentage reductions are presented in Table 5 indicating that the use of Z-DOM decreased the radiation exposure of the patients between 19% and 37% for the arterial phase and between 2% and 34% for the portal phase. The reason for difference in dose reduction rates observed for the two phases is based on the differences in the anatomic regions scanned. In the arterial phase, the scan area involves thorax and abdomen, while the portal phase includes abdominal and pelvic regions. Since the pelvic region with a bony structure has a higher radiation attenuation compared to the thoracic region filled with air, higher amounts of tube current are needed in this region. This explains why a lower dose reduction rate was observed in the portal phase compared to the arterial phase, especially for patients in group 2 and group 3.

On the other hand, Table 5 shows different rates of decreases observed in CTDI_vol_, DLP and E values across 3 groups of patients. Based on Table 5, it could be concluded that the rate of dose reduction was inversely proportional with the patient size. Dosimetric differences between the scan protocols were supported by statistical analysis, where all group based and phase based comparisons yielded significantly different results, except for the portal phase scan of patients in group 3. This was due to the high radiation attenuation property of the pelvic region in patients in group 3, so that, the tube current applied by Z-DOM was not much different from the routine protocol.

In a study that Lee et al. (18) conducted on abdominal CT scans, dose reduction up to 45% was reported and it was shown that higher dose reduction rates were achieved with lower body mass index. These results were parallel to the results obtained from our study. In another study carried out by S. Livingstone et al. (7) on contrast-enhanced biphasic abdominal examinations, dose reductions between 16% and 28% were achieved with the protocol using ATCM compared to fixed current protocols based on patient weight. According to the results of our study, dose reduction rates were observed approximately between 10% (group 3) and 35% (group 1) among the 3 groups when both phases were considered together (Table 5). The results of a comprehensive study in which dosimetric data from 12 centers in the USA were collected showed that the third quartile of the biphasic abdominal CT doses was 32 mSv (19). This value represented the exam specific reference dose level for these medical centers. In this study, the total E due to biphasic abdominal examination was reported as 20.8 mSv and 15.6 mSv, for routine protocol and ATCM protocol, respectively. These values showed that using ATCM techniques, Z-DOM in this case, provided CT scans with lower radiation doses.

In the second part of the study, the diagnostic quality of the images obtained by both protocols was compared using objective and subjective approaches. NI and CNR measurements and calculations were conducted as part of the objective image quality assessment. Table 6 shows the increase in NI values across all patient groups for the protocol using the Z-DOM technique. This increase was observed to become lower as the patient size increased. This is because the Z-DOM technique uses higher tube currents in overweight patients to maintain image quality at a certain level. Comparison of two protocols based on CNR values is given in Table 6. According to this table, CNR values regarding liver-fat and aorta-fat decreased between 5% and 19% in all patient groups for both arterial and portal phases. The conclusion reached with these two tables was that the use of Z-DOM technique leaded to lower objective image quality when compared to routine examination protocol. However, increased noise in the image and therefore decreased contrast between the tissues do not always mean that the image does not meet diagnostic standards required for a successful evaluation. Subjective image quality assessment performed in the last part of the study had an important role in this context. According to the results obtained from the subjective assessment which was made by a radiologist based on the scale given in Table 2, all images were concluded to have the criterion of acceptable diagnostic quality (Table 7).

It is of great importance that the patient dose is brought to the lowest possible level so as to present adequate diagnostic information to the clinician. In order to achieve this goal, it is necessary for the clinical staff to review all parameters of the examination protocols. On the other hand, further advances should follow in the present techniques of image reconstruction developed by the manufacturers which recently play a very important role in the dose reduction strategies. In addition, clinical physicists, technicians, and radiologists should have more detailed knowledge of the use of AEC techniques that are available for different scanners with different working principles.

### Study Limitations

The main limitation of our study was the lack of multiple subjective evaluations made on image quality. There was only one reader for image grading. Increasing the number of readers could help for less bias and more reliable results.

## Conclusion

In conclusion, when Z-DOM technique was used instead of weight-based fixed current protocol in contrast-enhanced biphasic abdominal examinations, it was observed that patient doses decreased inversely proportional to the patient size while keeping a sufficient image quality.

## Figures and Tables

**Table 1:**
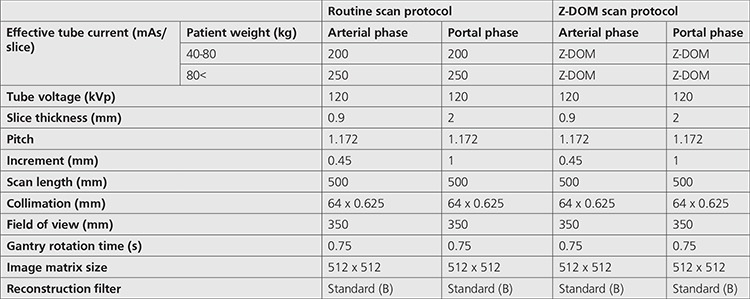
Contrast-enhanced biphasic abdominal scan protocols

**Table 2:**
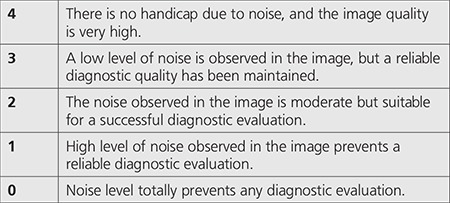
Grading scale for subjective evaluation

**Table 3:**
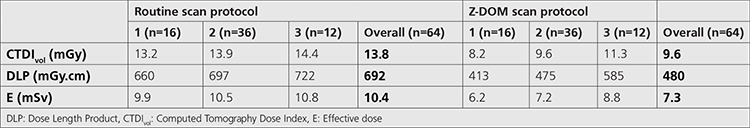
CTDI_vol_, DLP and E values of scan protocols for arterial phase

**Table 4:**
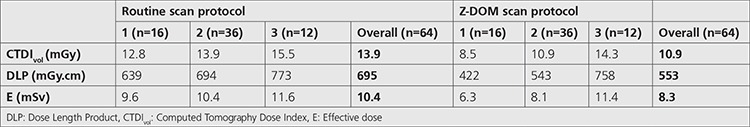
CTDI_vol_, DLP and E values of scan protocols for portal phase

**Table 5:**
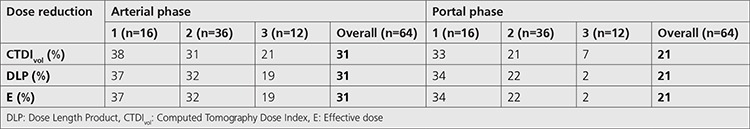
Dose reductions for patients in group 3

**Table 6:**
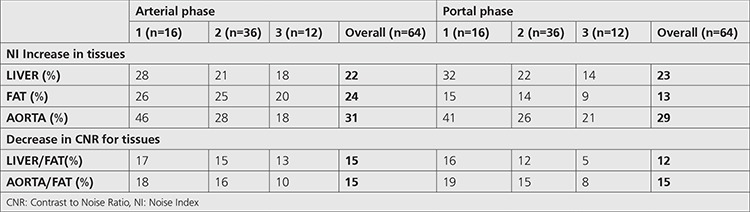
Group-specific and overall changing in Noise Index and Contrast to Noise Ratio due to the use of automatic tube current modulation protocol

**Table 7:**
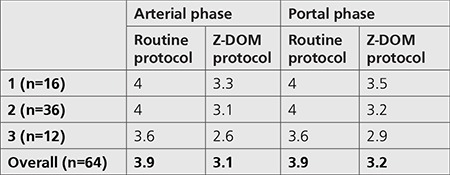
Mean values of subjective grading scores
